# County-Level Cervical Cancer Screening Coverage and Differences in Incidence and Mortality

**DOI:** 10.1001/jamanetworkopen.2025.26709

**Published:** 2025-08-13

**Authors:** Trisha L. Amboree, Jane R. Montealegre, Haluk Damgacioglu, Brian Orr, John Wrangle, Kalyani Sonawane, Ashish A. Deshmukh

**Affiliations:** 1 Department of Public Health Sciences, Medical University of South Carolina, Charleston; 2 Cancer Prevention & Control Program, Hollings Cancer Center, Medical University of South Carolina, Charleston; 3 Department of Behavioral Science, The University of Texas MD Anderson Cancer Center, Houston; 4 Department of Obstetrics and Gynecology–Gynecologic Oncology, Medical University of South Carolina, Charleston; 5 Department of Medicine–Hematology/Oncology, Medical University of South Carolina, Charleston; 6 Department of Pharmacology and Immunology, Medical University of South Carolina, Charleston

## Abstract

This cross-sectional study evaluates differences between cervical cancer incidence and mortality in counties with high vs low screening rates.

## Introduction

Recent research shows that cervical cancer incidence and mortality are 67% and 108% higher, respectively, in low-resourced US counties.^1^ County-level screening disparities may contribute to these differences,^2^ yet this remains unexplored. This study examines differences in cervical cancer incidence and mortality by county-level screening.

## Methods

For this cross-sectional study, we identified cervical cancer cases among women aged 20 years or older using the Surveillance, Epidemiology, and End Results (SEER)–22 database.^3^ Cervical cancer mortality was based on death certificate data ascertained by the National Center for Health Statistics. County-level cervical cancer screening was derived from SEER’s small area estimates^4^ for 1086 counties. Counties were considered to have repeatedly low vs high screening if they had less than 70% vs 80% or greater coverage during the 2011 to 2016 period and at least 1 earlier period (2008-2010 or 2004-2007). Counties not meeting these criteria were categorized as other. The less-than-70% threshold reflects coverage nearly 10 percentage points below the national target (79.2%) and below the current national average (74%-78%). The 80%-or-greater threshold exceeds both benchmarks, indicating consistently high coverage. Using SEER*Stat version 8.4.4 (National Cancer Institute), we estimated age-adjusted 5-year incidence and mortality rates for years 2016 to 2021 (excluding 2020 due to potential reporting biases from the COVID-19 pandemic^5^) and derived rate ratios (RRs) and 95% CIs to quantify differences in rates. County-level median household income (derived from American Community Survey 2017-2021 estimates) and metropolitan status (derived from 2013 Rural-Urban Continuum Codes, with 1-3 indicating urban and 4-9 indicating rural) were described.

This study followed the STROBE reporting guideline for cross-sectional studies. It was deemed exempt from review and the requirement for informed consent by the institutional review board at The Medical University of South Carolina because the data are deidentified and publicly available.

## Results

**Figure 1.**
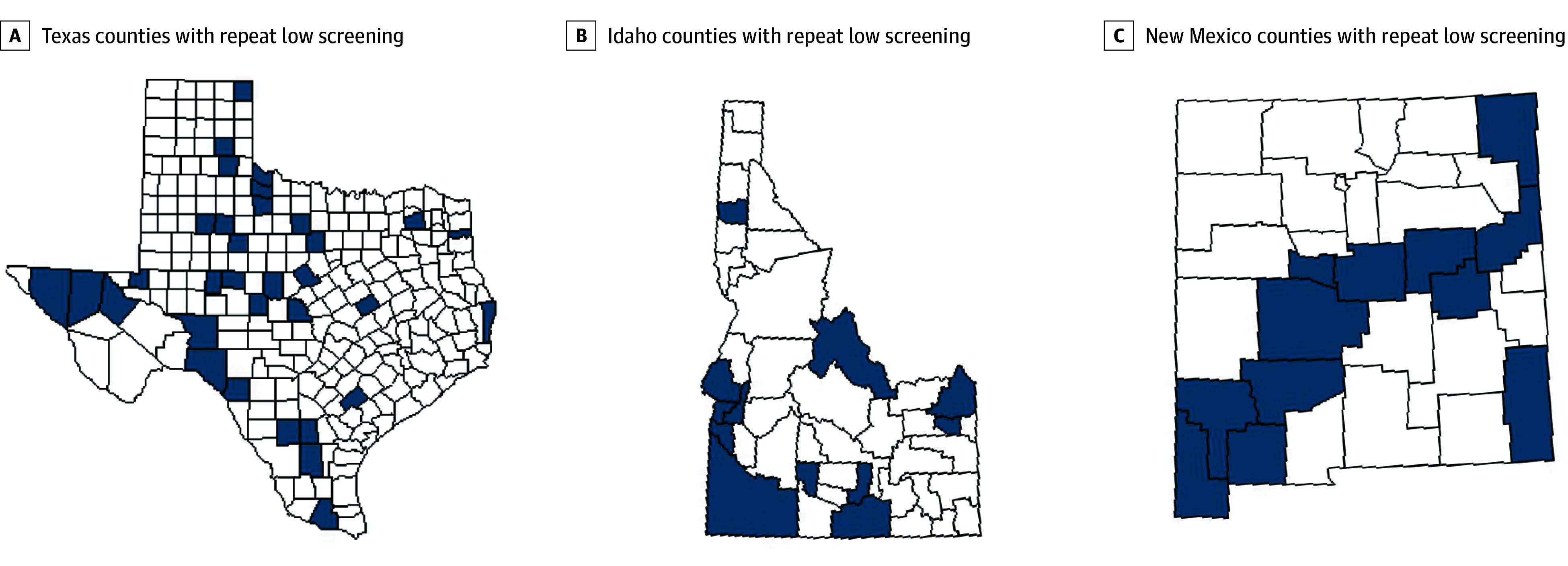
Three States With the Most Repeat Low Screening Coverage Counties: Texas, Idaho, and New Mexico Dark blue counties are those with repeatedly low (<70%) screening coverage. Texas counties (with the Federal Information Processing codes in parentheses) included Coke County (48081), Coleman County (48083), Comanche County (48093), Concho County (48095), Crockett County (48105), Culberson County (48109), DeWitt County (48123), Donley County (48129), Duval County (48131), Falls County (48145), Fisher County (48151), Foard County (48155), Garza County (48169), Hall County (48191), Hardeman County (48197), Hidalgo County (48215), Hopkins County (48223), Hudspeth County (48229), Kent County (48263), Kinney County (48271), Knox County (48275), La Salle County (48283), Lipscomb County (48295), Marion County (48315), McMullen County (48311), Newton County (48351), Palo Pinto County (48363), Reeves County (48389), San Saba County (48411), Sterling County (48431), Val Verde County (48465), Winkler County (48495), and Young County (48503). Idaho counties (with the Federal Information Processing codes in parentheses) included Benewah County (16009), Canyon County (16027), Cassia County (16031), Fremont County (16043), Gem County (16045), Gooding County (16047), Lemhi County (16059), Madison County (16065), Minidoka County (16067), Owyhee County (16073), Payette County (16075), and Washington County (16087). New Mexico counties (with the Federal Information Processing codes in parentheses) included De Baca County (35011), Grant County (35017), Guadalupe County (35019), Hidalgo County (35023), Lea County (35025), Luna County (35029), Quay County (35037), Sierra County (35051), Socorro County (35053), Torrance County (35057), Union County (35059), and Valencia County (35061).

A total of 70 counties were identified as repeat low coverage, 141 as repeat high coverage, and 875 as other. Most repeat low coverage counties were rural (61 [87.1%]), and all had a median household income of less than $75 000, while most repeat high coverage counties were urban (119 [84.4%]), and 72 (51.1%) had a median household income of $75 000 or greater. Most repeat low coverage counties were from Texas (33 [47.1%]), Idaho (12 [17.1%]), and New Mexico (12 [17.1%]) (Figure 1).

**Figure 2.**
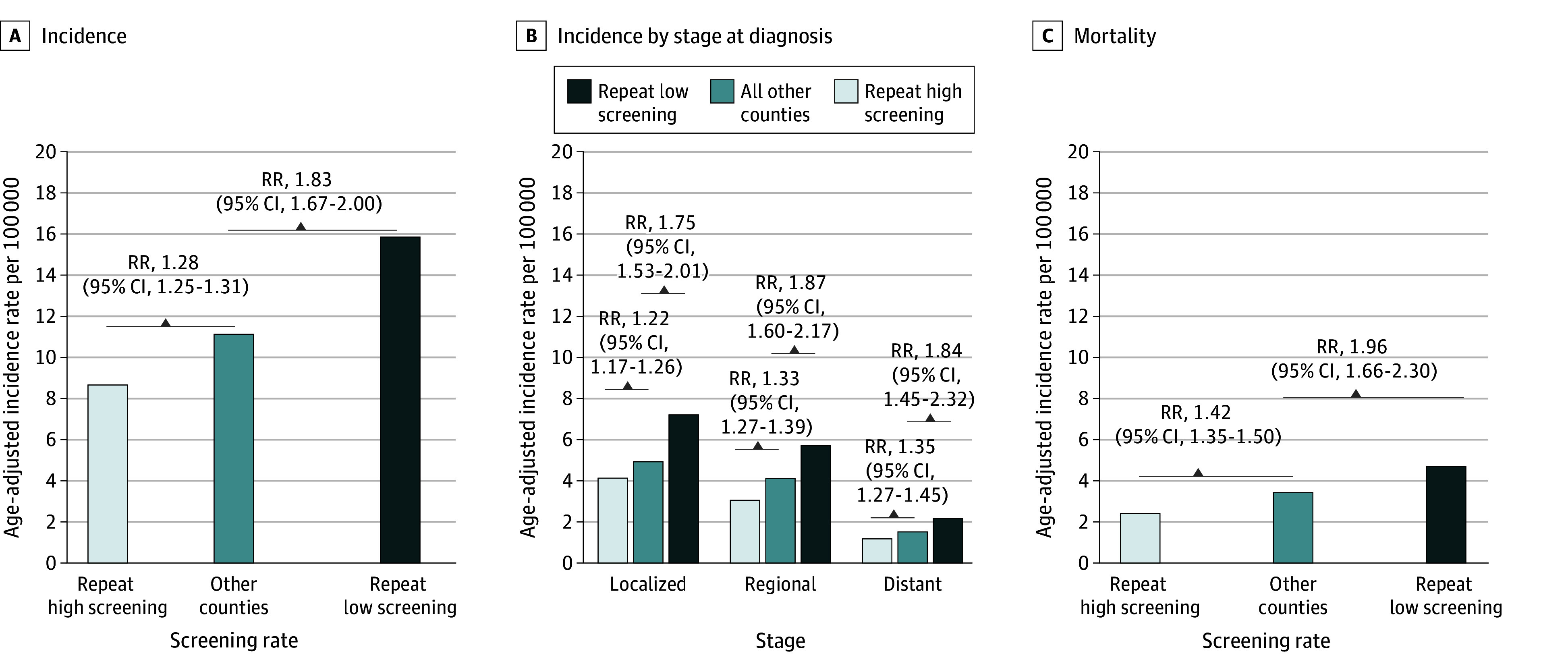
Cervical Cancer Incidence, Stage at Diagnosis, and Mortality by County-Level Screening Coverage The states included in the Surveillance, Epidemiology, and End Results–22 database are Alaska, California, Connecticut, Georgia, Hawaii, Idaho, Illinois, Iowa, Kentucky, Louisiana, Massachusetts, New Jersey, New Mexico, New York, Texas, Utah, and Washington. Cervical cancer cases were identified using *International Classification of Diseases Oncology*, *Third Edition*, codes C53.0 to C53.9 and histology codes 8010 to 8671 and 8940 to 8941. All rates were age-adjusted to the 2000 US standard population. RR indicates rate ratio.

During the 2016 to 2021 period, cervical cancer incidence was 28% (RR, 1.28 [95% CI, 1.25-1.31]) and 83% (RR, 1.83 [95% CI, 1.67-2.00]) higher in other and repeat low coverage counties, respectively, compared with high coverage counties (Figure 2A). When stratified by stage at diagnosis, localized-stage incidence was 22% (RR, 1.22 [95% CI, 1.17-1.26]) and 75% (RR, 1.75 [95% CI, 1.53-2.01]) higher, regional-stage was 33% (RR, 1.33 [95% CI, 1.27-1.39]) and 87% (RR, 1.87 [95% CI, 1.60-2.17]) higher, and distant-stage was 35% (RR, 1.35 [95% CI, 1.27-1.45]) and 84% (RR, 1.84 [95% CI, 1.45-2.32]) higher in other and repeat low coverage counties, respectively, vs high coverage counties (Figure 2B). During the 2016 to 2021 period, cervical cancer mortality was 42% (RR, 1.42 [95% CI, 1.35-1.50]) and 96% (RR, 1.96 [95% CI, 1.66-2.30]) higher in other and repeat low coverage counties, respectively, vs high coverage counties (Figure 2C).

## Discussion

We report nearly twofold higher cervical cancer incidence, late-stage diagnosis, and mortality in counties with repeatedly low vs high cervical cancer screening coverage. Most counties with repeat low coverage were rural and lower income. These findings help elucidate previously reported differences in cervical cancer incidence and mortality in rural and lower-income counties.^1,6^ Additionally, our study pinpoints specific counties in Texas, Idaho, and New Mexico with repeatedly low coverage, offering crucial data to improve targeted screening efforts.

Certain limitations should be considered when interpreting these results. First, this study’s cross-sectional design precludes causal inference. Second, we did not adjust for county-level sociodemographic factors (eg, income, rurality), which may influence both screening rates and cervical cancer outcomes. As such, observed differences between high and low screening counties may reflect underlying socioeconomic disparities in addition to differences in screening. Additionally, there are potential biases associated with self-reported screening measures.

Our study findings underscore the urgent need to improve cervical cancer screening in rural and lower-income counties. Particularly, counties where screening coverage is repeatedly low should be targeted.

## Notes

Supplement.
Data Sharing Statement
