# Supplementary material for: County-Level Cervical Cancer Screening Coverage and Differences in Incidence and Mortality
Source: JAMA Netw Open. 2025 Aug 13;8(8):e2526709. doi: 10.1001/jamanetworkopen.2025.26709 (PMC12351409; doi:10.1001/jamanetworkopen.2025.26709)
Supplement: Supplement. — Data Sharing Statement [file jamanetwopen-e2526709-s001.pdf]

Data Sharing Statement

Amboree. County-Level Cervical Cancer Screening Coverage and Differences in Incidence and Mortality. *JAMA Netw Open*. Published August 13, 2025. doi:10.1001/jamanetworkopen.2025.26709

Data
Data available: No

Additional Information
Explanation for why data not available: Data for the Surveillance, Epidemiology, and End Results (SEER) database are publicly available at https://seer.cancer.gov/data/.
